# Artificial Intelligence in the Diagnosis of Odontogenous Cysts and Ameloblastomas—A Systematic Review and Meta-Analysis

**DOI:** 10.3390/jcm15062447

**Published:** 2026-03-23

**Authors:** Anna Takács, Dalma Tábi, Bianca Golzio Navarro Cavalcante, Bence Szabó, Alexander Schulze Wenning, Gábor Gerber, Péter Hermann, Gábor Varga, Péter Hegyi, Márton Kivovics

**Affiliations:** 1 Department of Public Dental Health, Semmelweis University, Szentkirályi u. 40., 1088 Budapest, Hungary; 2 Centre for Translational Medicine, Semmelweis University, Baross u. 22., 1085 Budapest, Hungary; 3 Department of Oral Biology, Semmelweis University, Nagyvárad tér 4., 1089 Budapest, Hungary; 4 Department of Anatomy, Histology and Embryology, Semmelweis University, Tűzoltó u. 58., 1094 Budapest, Hungary; 5 Department of Prosthodontics, Semmelweis University, Szentkirályi u. 47., 1088 Budapest, Hungary; 6 Institute for Translational Medicine, Szentágothai Research Centre, Medical School, University of Pécs, Szigeti út 24., 7624 Pécs, Hungary; 7 Division of Pancreatic Diseases, Heart and Vascular Center, Semmelweis University, 1083 Budapest, Hungary

**Keywords:** artificial intelligence, convolutional neural networks, cysts, ameloblastoma, diagnosis, meta-analysis, review

## Abstract

**Background/Objectives**: Odontogenic cysts and ameloblastomas (AB) are mostly asymptomatic, often discovered later due to severe symptoms, and only histopathological examination provides definitive diagnosis. AI-assisted diagnostics offer a fast, noninvasive, painless diagnostic tool. To our knowledge, this is the first meta-analysis aiming to evaluate the classification, detection, and segmentation performance of artificial intelligence (AI) for odontogenic cysts and ABs as distinct entities and to determine if it can achieve clinically acceptable accuracy. **Methods**: Our systematic search was conducted on 11 January 2026, in Medline, EMBASE, and Cochrane Central Register of Controlled Trials without restrictions or filters. Studies comparing AI diagnostics with histopathological diagnostics for odontogenic cysts and ABs were included. Diagnostic parameters, including sensitivity, specificity, and accuracy, were extracted and analyzed; additionally, diagnostic odds ratios were calculated. Risk of bias was assessed using the Quality Assessment of Diagnostic Accuracy Studies (QUADAS-2) tool. Recommendations of the GRADE workgroup were followed to determine the certainty of evidence. **Results**: Thirteen articles were found eligible, of which seven were included in our meta-analysis. The group with the highest sensitivity (Se) was the “no lesion” (N) group (0.9726, 95% CI 0.9284–1; I2 = 46%), followed by the radicular cyst (RC) (mean 0.9054, 95% CI 0.8051–1; I2 = 89%), dentigerous cyst (DC) (mean 0.8788, 95% CI 0.7828–0.9749; I2 = 93%), odontogenic keratocyst (OKC) (0.763, 95% CI 0.6999–0.8262; I2 = 14%) and AB (mean 0.4369, 95% CI 0.231–0.6429; I2 = 79%) groups. Results for AB, RC, and DC were statistically significant. The AB achieved the highest specificity (Sp) (mean 0.9889, 95% CI 0.9736–1; I2 = 0%), followed by RC (mean 0.9724, 95% CI 0.9431–1; I2 = 79%), DC (mean 0.9516, 95% CI 0.9116 0.9917; I2 = 90%), N (mean 0.9226, 95% CI 0.8385–1; I2 = 95%) and OKC (mean 0.8991, 95% CI 0.8683–0.9298; I2 = 8%) groups. DC, N, and RC had statistically significant results. Diagnostic odds ratios (DOR) showed that classification was better than chance for all lesion types. **Conclusions**: AI demonstrated high specificity, and is therefore effective in identifying healthy individuals. However, its sensitivity in detecting diseased patients remains suboptimal and requires further improvement.

## 1. Introduction

AI algorithms revolutionize modern healthcare. In dentistry, key applications include diagnostic support, personalized treatment planning (such as orthodontics or implantology), and risk analysis for conditions such as oral cancer, caries, and periodontitis [1].

In dental diagnostics and imaging, convolutional neural networks (CNNs) were inspired by the visual cortex of the brain [2]. Their exceptional ability to analyze spatial information has resulted in high diagnostic accuracy across various areas, including oral cancer detection and classification [3], caries diagnostics [4], and periodontology [5].

Odontogenic cysts are common lesions in the oral cavity (13.8% prevalence) [6]. Ameloblastomas (AB), although benign, are locally aggressive and account for 10% of odontogenic tumors, affecting 0.5 per million people per year worldwide [2].

They are mostly asymptomatic, often discovered incidentally or later on due to severe symptoms such as tooth displacement, malocclusion, facial asymmetry, or pathological fractures. The possibility of malignant transformation has also been described in the literature [3].

Despite severe complications, a definitive diagnosis can only be made after an invasive and time-consuming histopathological examination of the lesion removed.

As cysts and ABs are usually visible on panoramic radiographs (OPGs), CNN analysis provides a new diagnostic solution for early detection (the ability to recognize the presence of a particular object), classification (the ability to predefine grouping of the detected object) and segmentation (identification of pixels belonging to the detected structure) of lesions that occur at a stage where treatment is easier and severe complications can be avoided [7].

Meta-analyses support the continued use of AI methods, as the measured classification accuracy, sensitivity (Se), and specificity (Sp) ranged between 8.0 and 0.9 [7,8].

Data on individual cyst types or AB are fundamental for the validation of AI tools in clinical practice. However, these studies focused on a pooled set of cysts and did not assess the classification performance for each lesion type separately.

Research on detection [9] and segmentation [10] is promising, but still limited, with no meta-analysis available to date.

We aim to provide a comprehensive overview of recent advancements in classification, detection, and segmentation performance. In addition, we aim to verify whether artificial intelligence (AI) diagnostics can achieve clinically acceptable accuracy.

## 2. Materials and Methods

This systematic review and meta-analysis followed the PRISMA 2020 guideline [11] (Supplementary Materials—Table S1) and the Cochrane Handbook [12]. We adhered to the previously registered protocol (registration number CRD42024523372) with minor exceptions: in the meta-analysis part, positive predictive value (PPV) and negative predictive value (NPV) could not be evaluated due to missing data. We could only conduct a systematic review for detection performance. For this assessment, only Se and PPV could be incorporated from the predefined primary outcomes; however, average precision (AP) and F1 score were additionally included. Sample size weighted averages were calculated to reflect the trends reported in each study.

### 2.1. Eligibility Criteria

Using the PIRD (Population, Index test, Reference test, Diagnosis of interest) framework, we included patients with panoramic radiographs (P). AI diagnostics (I) were compared to histopathological diagnostics (R) to assess their accuracy in diagnosing odontogenic cysts and AB (D). Diagnostic performance was assessed using Se and Sp, the area under the receiver operating characteristic curve (AUC), and positive and negative predictive values.

#### Inclusion and Exclusion Criteria

Diagnostic studies using panoramic radiographs were included if they reported data for any lesion type separately. Diagnoses had to be based on histopathological examination.

Reviews and studies with different imaging techniques (e.g., CBCT and periapical radiographs) were excluded.

### 2.2. Information Sources

We conducted a systematic search on 11 January 2026, in Medline, EMBASE, and Cochrane Central Register of Controlled Trials, without any restrictions or filters. In addition, the citation and reference lists of included articles were checked manually. Our search strategy revolved around cyst types, “ameloblastoma” and “artificial intelligence” (Table 1).

### 2.3. Selection Process

The EndNote X9 (Clarivate Analytics, Philadelphia, PA, USA) reference management software was used. First, duplicates were removed. Subsequently, two independent authors (A.T. and D.T.) screened articles by title and abstract, then by full text. For both steps, interrater reliability was evaluated using Cohen’s kappa coefficients. Disagreements were resolved by a third independent investigator (M.K.). Study authors were contacted when full texts were unavailable.

### 2.4. Data Collection Process

Two authors (A.T. and D.T.) collected data individually in a standardized form (Excel [Microsoft Corporation, Redmond, WA, USA] data sheet). Disagreements were resolved by a third independent investigator (M.K.). Study authors were contacted in cases of missing or unclear data.

### 2.5. Data Items

The following data were extracted: first author, year of publication, type of lesion, AI system and its structure, number of panoramic radiographs per group, and outcomes.

The statistical models of AI were classified according to their mathematical structure. All included articles used neural networks and deep learning models belonging to CNNs.

Primary outcomes included Se, Sp, AUC, and positive and negative predictive values. The numbers or ratios of true positive (TP), false positive (FP), true negative (TN), and false negative (FN) results were also collected when available.

Groups were formed according to lesion types available: AB, RC, DC, and OKC. As numerous articles tended to include a “No lesion group”, we also adapted it into our analysis as group N.

### 2.6. Study Risk of Bias Assessment

For each study, patient selection, index test, reference standard, and the flow and timing were assessed across four domains of the Quality Assessment of Diagnostic Accuracy Studies (QUADAS-2) tool. All domains for risk of bias, as well as the first three for applicability concerns, could be described as “low,” “unclear,” or “high” [13].

Two independent examiners (A.T. and D.T.) conducted the assessment, and disagreements were resolved through a third independent author (M.K.).

### 2.7. Synthesis Methods

For the classification performance outcome, random-effect meta-analyses were fitted for each lesion type with at least three available studies that included either true positive, true negative, false positive and false negative numbers or a point and interval estimate of specificity and sensitivity. Specificity and sensitivity were estimated with 95% confidence intervals (CI), using a random intercept logistic regression model as recommended by Schwarzer et al. [14] and Stijnen et al. [15]. The maximum likelihood method was used to estimate the measure of heterogeneity variance (τ2). The Clopper–Pearson method [16] was used to estimate CIs of each study.

We plotted the individual and pooled sensitivities and specificities of the included studies, their summary estimates, and the corresponding 95% confidence and prediction regions on forest plots.

PPV and NPV results were calculated from the estimated specificity and sensitivity values at a prevalence rate of 30%. The equations for these calculations are $PPV=\frac{Sensitivity\times Prevalence}{Sensitivity\times Prevalence+\left(1-Specificity\right)\times (1-Prevalence)}$ and $NPV=\frac{Specificity\times (1-Prevalence)}{Specificity\times \left(1-Prevalence\right)+\left(1-Sensitivity\right)\times Prevalence}$.

The diagnostic odds ratio (DOR) with its 95% CI was also calculated, which is a single indicator that combines sensitivity and specificity of a diagnostic test, thus simplifying the comparison of test performance. It is defined as the ratio of odds of a positive test result for individuals with the disease to the odds of a positive test result in individuals without the disease. The DOR ranges from 0 to infinity, with higher values indicating better performance [17].

Heterogeneity was assessed by calculating I^2^ measure and its confidence interval arising from separate univariate analyses.

For each lesion type, we also estimated a pooled random-effect meta-ROC curve with a 95% confidence region using the non-parametric approach in the nsROC package [18], which implemented the methodology proposed by Martinez-Camblor et al. [19].

Statistical analyses were carried out with R statistical software (version 4.1.2., R-core team, 2023) [20] using the meta [21] and the lme4 [22] packages, based in part on the web-tool of Freeman et al. [23]. Statistical analyses followed the advice of Harrer et al. [24].

For detection performance outcome, the criteria for a meta-analysis were not fulfilled. Instead of a meta-analysis, a summary plot was created to visualize the results reported in the studies. For the PPV, sensitivity, F1-score and AP, a simple, sample size weighted average was plotted for each lesion type to better show the trends reported in each study.

### 2.8. Certainty of Evidence

The recommendations of the “Grades of Recommendation, Assessment, Development, and Evaluation (GRADE)” workgroup were followed to assess the certainty of evidence [25]. Two reviewers (A.T. and D.T.) conducted the evaluation individually. A third independent investigator (M.K.) resolved disagreements.

## 3. Results

### 3.1. Search and Selection

Altogether, the systematic search identified 5664 articles. After duplication removal, 4808 studies were screened for title and abstract, and 33 were found eligible. Finally, 11 articles were included after the full-text selection [9,10,26,27,28,29,30,31,32,33,34]. Twenty articles had to be excluded because of their different study designs [35,36,37,38,39,40,41,42,43,44,45,46,47,48,49,50,51,52,53,54] and two because of missing histopathological diagnoses [55,56].

**Figure 1:**
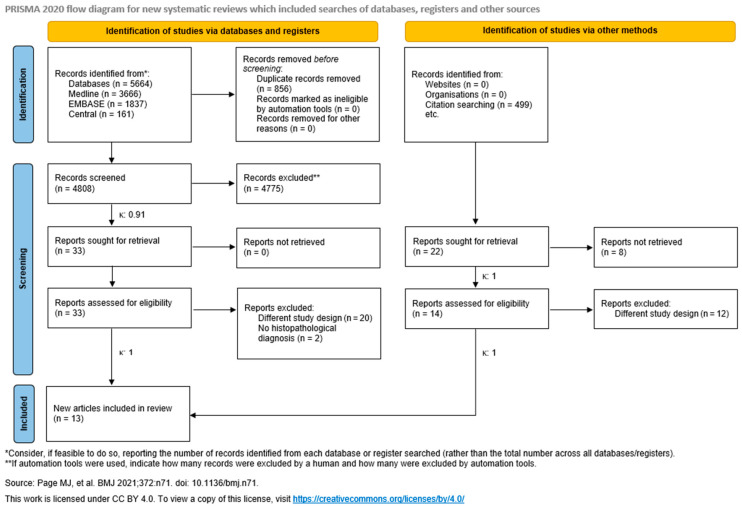
PRISMA flow chart [11].

Citations and references were also searched. Of 499 articles, 21 were searched for retrieval. Seven reports could not be retrieved [57,58,59,60,61,62,63] and 12 had different designs [64,65,66,67,68,69,70,71,72,73,74,75]. Finally, two more studies could be added to our review [76,77] (Figure 1).

Seven articles included confidence intervals (CI) or confusion matrixes with TP, TN, FP, and FN data, allowing only a meta-analysis of classification performance. Detection and segmentation were addressed in the systematic review.

### 3.2. Basic Characteristics of Included Studies

The baseline characteristics of the included studies can be found in Supplementary Materials—Table S2.

### 3.3. Classification—Meta-Analysis

#### 3.3.1. Sensitivity (Se)

**Figure 2:**
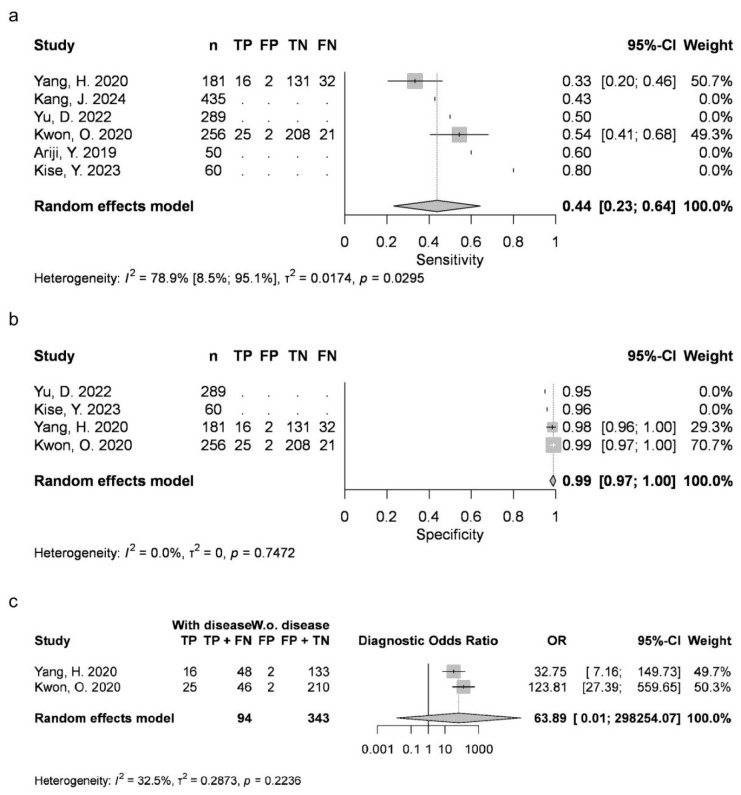
AB classification sensitivity (**a**), specificity (**b**) and diagnostic odds ratio (**c**) [9,10,26,27,34,76].

**Figure 3:**
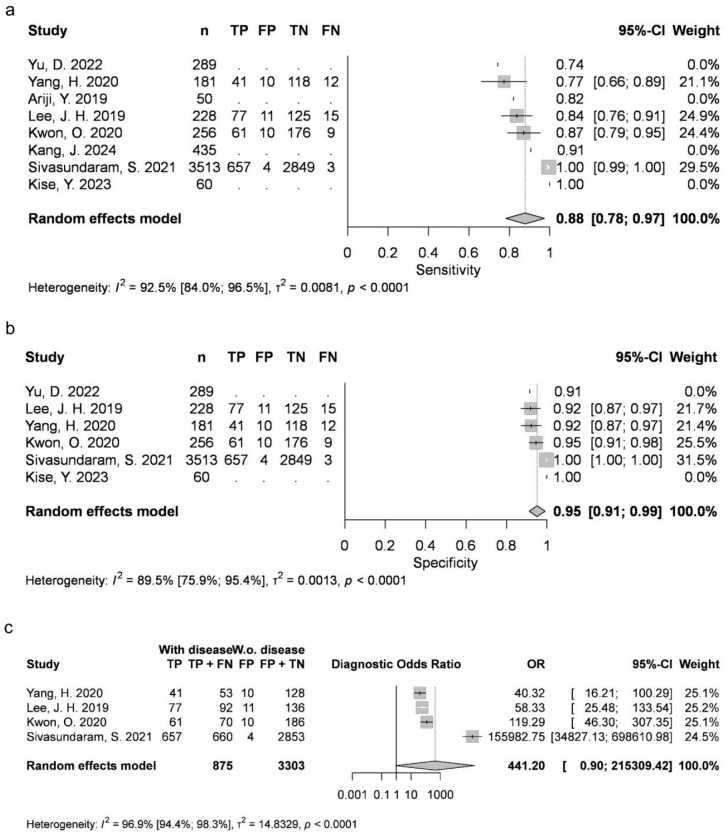
DC classification sensitivity (**a**), specificity (**b**) and diagnostic odds ratio (**c**) [9,10,26,27,29,34,76,77].

**Figure 4:**
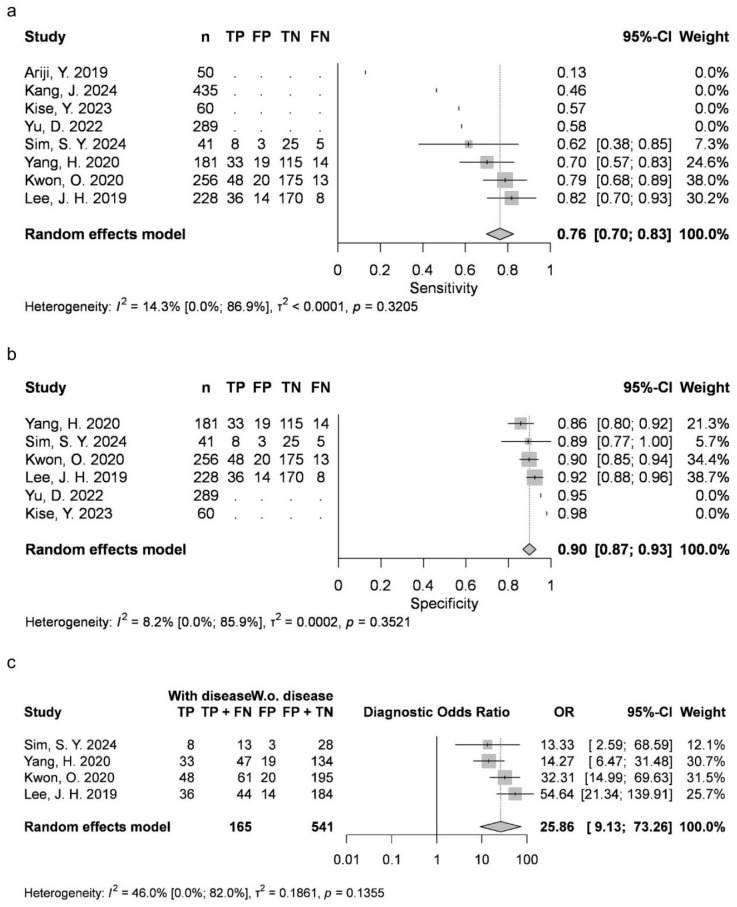
OKC classification sensitivity (**a**), specificity (**b**) and diagnostic odds ratio (**c**) [9,10,26,27,29,31,34,76].

**Figure 5:**
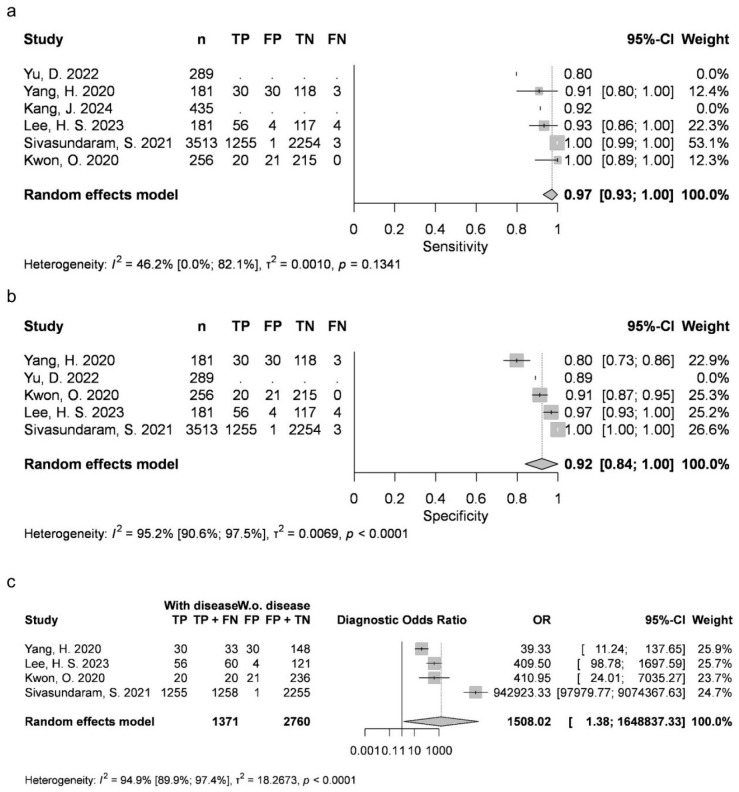
N classification sensitivity (**a**), specificity (**b**) and diagnostic odds ratio (**c**) [9,10,28,34,76,77].

**Figure 6:**
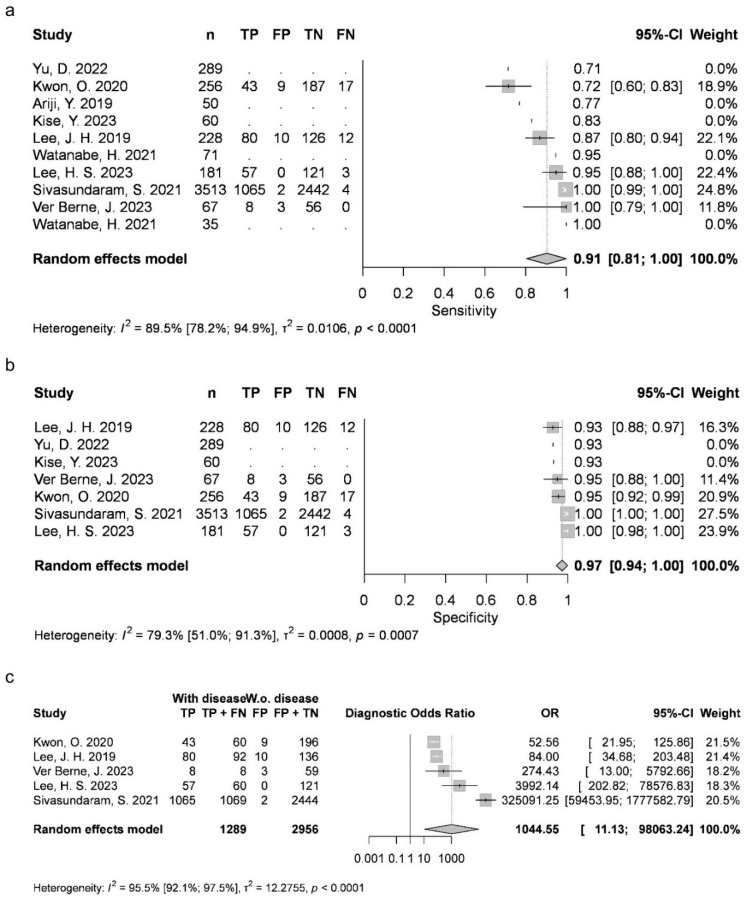
RC classification sensitivity (**a**), specificity (**b**) and diagnostic odds ratio (**c**) [9,10,26,27,28,29,32,33,77].

Two articles provided data for AB (mean 0.4369, 95% CI 0.231–0.6429; I2 = 79%). Four studies were available for DC (mean 0.8788, 95% CI 0.7828–0.9749; I2 = 93%), OKC (0.763, 95% CI 0.6999–0.8262; I2 = 14%), and N groups (0.9726, 95% CI 0.9284–1; I2 = 46%). Five articles were included for RC (mean 0.9054, 95% CI 0.8051–1; I2 = 89%). AB, RC and DC had statistically significant results. Results were clinically significant for each group, except for AB (Figure 2, Figure 3, Figure 4, Figure 5 and Figure 6).

#### 3.3.2. Specificity (Sp)

Two articles reported measurements for AB (mean 0.9889, 95% CI 0.9736–1; I2 = 0%), with four articles for DC (mean 0.9516, 95% CI 0.9116–0.9917; I2 = 90%), OKC (mean 0.8991, 95% CI 0.8683–0.9298; I2 = 8%) and N (mean 0.9226, 95% CI 0.8385–1; I2 = 95%), as well as five articles for RC (mean 0.9724, 95% CI 0.9431–1; I2 = 79%). DC, N and RC had statistically significant results. All groups achieved clinically significant values (Figure 2, Figure 3, Figure 4, Figure 5 and Figure 6).

#### 3.3.3. Diagnostic Odds Ratio (DOR)

Two articles about AB could be included (mean 63.8918, 95% CI 0.0137–298,254.066; I2 = 32%), with four about DC (mean 441.1951, 95% CI 0.9041–215,309.4163; I2 = 97%), OKC (mean 25.862, 95% CI 9.1302–73.2566; I2 = 46%) and N (mean 1508.0169, 95% CI 1.3792–1,648,837.3325; I2 = 95%), as well as five about RC (mean 1044.5518, 95% CI 11.1264–98,063.2391; I2 = 96%). DC, N and RC had statistically significant results (Figure 2, Figure 3, Figure 4, Figure 5 and Figure 6).

#### 3.3.4. Area Under the Curve (AUC)

Receiver operating characteristic (ROC) curves were generated for each group. The AUC was 0.714 for AB, 0.901 for DC, 0.93 for N, 0.823 for OKC and 0.959 for RC (Supplementary Materials—Figure S1).

### 3.4. Systematic Review

#### 3.4.1. Detection

##### Positive Predictive Value

Four articles provided information on PPV. Kwon et al. measured the total PPV of 0.78 from DC, RC, OKC, AB, and N images [9]. Yu et al. published 0.6132 for DC, 0.575 for RC, 0.4988 for AB, and 0.5119 for OKC, with a total PPV of 0.5497 [10]. Watanabe et al. measured PPV in two groups: the first dataset had a PPV of 0.886 for RC and 0.933 for the pooled DC, OKC, and nasopalatine duct cyst (NPDC) group, and the overall PPV was 0.898. In the second dataset, the PPV for RC was 0.87, and 1 in the pooled group of DC, OKC, and NPDC, so the total PPV was 0.9 [33]. Rašić et al. measured a PPV of 0.858 for RC [30].

Considering these results, we calculated the sample size weighted averages for each lesion type: 0.4988 for AB, 0.69 for DC, 0.96 for OKC, 0.83 for RC, and 0.7 in total (Supplementary Materials—Figure S2).

##### Sensitivity

There were seven articles about Se. Kwon et al. measured the total Se of 0.74 from DC, RC, OKC, AB, and N images [9]. Ariji et al. found an Se value of 0.71 for AB, 1 for OKC, 0.88 for DC, and 0.81 for RC [26]. Yu et al. published 0.7236 for DC, 0.6349 for RC, 0.5112 for AB, and 0.6337 for OKC, with a total Se of 0.6259 [10]. Kise et al. measured 0.71 for AB, 0.88 for OKC, 1 for DC, 0.75 for RC, and 0.92 for Stafne cyst, and the overall value was 0.87 [27]. Watanabe et al. measured Se in two groups: in the first dataset, 0.78 for RC and 0.667 for the pooled group of DC, OKC, and NPDC, ending with a total Se of 0.746. In the second dataset, the Se for RC was 0.8 and 0.7 for the pooled group of DC, OKC, and NPDC, so the overall Se value was 0.771 [33]. Kang et al. measured an Se value of 0.893 for AB, 0.814 for OKC, and 0.917 for DC [76]. Rašić et al. measured an Se value of 0.667 for RC [30].

Considering these results, we calculated the sample size weighted averages for each lesion type: 0.82 for AB, 0.88 for DC, 0.83 for OKC, 0.71 for RC, and 0.71 in total (Supplementary Materials—Figure S2).

##### F1 Score

Two articles assessed the F1 score. Kwon et al. measured the total F1 score of 0.76 from DC, RC, OKC, AB, and N images [9]. Watanabe et al. measured the F1 score in two groups: in the first dataset, 0.83 for RC and 0.778 for the pooled DC, OKC, and NPDC group, ending with a total mean of 0.815. In the second dataset, F1 for RC was 0.833 and 0.824 for the pooled DC, OKC, and NPDC group, so the overall mean value was 0.831 [33].

Considering these results, we calculated the sample size weighted averages for available lesion types: 0.79 for DC, 0.79 for OKC, 0.83 for RC, and 0.78 in total (Supplementary Materials—Figure S2).

##### Average Precision

Three studies measured AP. Kwon et al. reported an AP of 0.91 for DC, 0.79 for RC, 0.67 for OKC, 0.78 for AB, and a pooled mean of 0.79 with a standard deviation of 0.12 [9]. Yu et al. published 0.7202 for DC, 0.6954 for RC, 0.6543 for AB, and 0.6432 for OKC and a pooled mean of 0.6783 [10]. Ver Berne et al. measured 0.83 AP for RC and 0.74 for periapical granulomas [32].

Considering these results, we calculated the sample size weighted averages for each lesion type: 0.76 for AB, 0.84 for DC, 0.67 for OKC, 0.77 for RC, and a mean of 0.73 altogether (Supplementary Materials—Figure S2).

#### 3.4.2. Segmentation

Only two articles investigated segmentation. The Se of the DL CNN system of Yu et al. was 0.7327 for DC, 0.6751 for RC, 0.5135 for AB, and 0.6422 for OKC, and the average was 0.6409. The Sp was 0.7142 for DC, 0.7253 for RC, and 0.689 for OKC, with an overall mean of 0.7064. Pixel accuracy was 0.7132 for DC, 0.6843 for RC, 0.6725 for AB, and 0.6542 for OKC, and the average was 0.6811. The intersection over union (IoU) was 0.7326 for DC, 0.7234 for RC, 0.6754 for AB, and 0.7023 for OKC, and the mean was 0.7084 [10].

Sivasundaram et al. published data about a modified LeNet CNN. They measured an Se of 0.987 for DC, 0.989 for RC, 0.988 for “odontogenic cyst”, and 0.988 in total. The Sp was 0.987 for DC, 0.989 for RC, 0.988 for “odontogenic cyst”, and 0.988 in total. Pixel accuracy was 0.985 for DC, 0.976 for RC, 0.984 for “odontogenic cyst,” and 0.985 in total. The negative predictive value was 0.976 for DC, 0.978 for RC, 0.971 for “odontogenic cyst”, and 0.9783 in total. The positive predictive value was 0.976 for DC, 0.971 for RC, 0.971 for “odontogenic cyst”, and 0.972 in total. The IoU was 0.973 for DC, 0.981 for RC, 0.976 for “odontogenic cyst,” and 0.976 in total. The dice similarity coefficient (DSC) was 0.987 for DC, 0.976 for RC, 0.989 for odontogenic cyst, and 0.9804 in total [77].

### 3.5. Risk of Bias Assessment

We used the QUADAS-2 tool for our analysis. Most articles indicated a low risk of bias in the domains “index test,” “reference standard,” and “flow and timing.” In the “patient selection” domain, seven studies showed a low risk of bias, five were classified as unclear, and one had a high risk of bias [77]. Only one high-risk article could be found in both the “index test” [28] and “flow and timing” [76] domains. There were no articles with high-risk concerns in any of the applicability domains. For both the “index test” and “reference standard,” only one study had an unclear risk, while all articles in the “patient selection” domain were assessed as low-risk. The results are presented in Supplementary Materials—Figure S3.

### 3.6. Publication Bias and Heterogeneity

Publication bias was evaluated through funnel plots. They appeared symmetrical, indicating a low risk of publication bias. However, due to the low number of articles analyzed, these results should be interpreted with caution (Supplementary Materials—Figure S4).

Heterogeneity was high for all lesion types except for OKC. This result can be attributed to the variations of AI models used in the analysis and the differences in training and image augmentation.

### 3.7. Certainty of Evidence Assessment

Most studies had moderate certainty of evidence. The only exception was AB, which was rated very low due to a lack of a large effect. Risk of bias and “indirectness” were mostly not considered serious, while “inconsistency” and “imprecision” tended to be serious. Other factors that affected this were undetected publication bias and the large magnitude of effect (Supplementary Materials—Figure S5).

## 4. Discussion

This article aimed to assess the current knowledge on the accuracy of AI diagnostics of cysts and AB in OPGs. Due to the lack of existing literature, a meta-analysis was feasible only for assessing classification performance.

The mean Se and Sp for RC and N were both above 0.9. The Sp of DC was high, and the mean Se 0.8788 was nearing the 0.9 value. In the OKC group, none of the mean values reached 0.9, but were very close to it: the Se was 0.763, and the Sp was 0.8991. The assessment of AB was limited to two articles that reported low Se, but an Sp value exceeding 0.9.

Establishing a minimally acceptable threshold for both Se and Sp may be challenging [78]. Power et al. consider a test to be effective if Se + Sp is at least 1.5, where 2 is perfect, and 1 is useless. This narrative shows that the test was beneficial in all groups except AB [79].

Leeflang et al. noted that the sensitivity and specificity of AI should match or exceed those of other existing methods [78].

The only noninvasive diagnostic alternative is human examination. Yang et al. found that AI diagnostics were at least comparable to expert dentist opinions [34]. In their study, Cardoso et al. examined the classification performance of individuals with varying levels of expertise in interpreting OPGs. They reported a mean Se of 0.6133 and a mean Sp of 0.86 for AB, a mean Se of 0.84 and mean Sp of 0.88 for DC, and a mean Se of 0.6267 and a mean Sp of 0.8 for OKC. Comparing these results to ours, we can see that AI was superior in all groups and all measurements, except for the Se of AB [80].

Some studies did not report confusion matrices or CI. They were, therefore, inappropriate for our statistical analysis. However, their results were consistent with ours.

None of the prior meta-analyses separately evaluated the classification performance for odontogenic cysts or AB.

Although Shrivastava et al. grouped simple bone cysts, Stafne bone cysts, and glandular odontogenic cysts with odontogenic cysts within OPGs, their Se of 0.93 (95% CI 0.77–0.98) and Sp of 0.93 (95% CI 0.83–0.97) were consistent with our results [7].

Fedato Tobias et al. also reported high diagnostic accuracy with the pooled dataset of odontogenic OKC and AB [8]. Although our analysis indicated a lower performance for AB, when pooled with the OKC group, the results were consistent.

DOR exceeded 1 in all cases, indicating that each test performed better than chance. Higher values signify superiority in distinguishing those with and without the given condition. Of the groups, N achieved the most impressive result, followed by RC, DC, and AB, while OKC recorded the lowest DOR.

ROC curves were generated to assess the AUC. According to Šimundić et al., the diagnostic accuracy of a test is considered “excellent” if the AUC is between 0.9 and 1, “very good” if it is between 0.8 and 0.9, and “good” if the value is between 0.7 and 0.8 [81]. In this analysis, DC, RC, and N were rated as “excellent,” OKC as “very good,” and AB as “good.” It is important to note that the number of articles included was low, and their results were clustered in a similar area—the upper left corner—of the plot, which indicates a cautious interpretation.

Detection performance could be illustrated with the help of Se.

For AB, measured values ranged from 0.5112 to 0.893. The calculated sample size weighted mean was 0.82. For OKC detection, data from 0.6337 to 1 were published. The sample size weighted mean was 0.83. In the RC group, the Se was 0.6349–0.81, and the weighted mean was 0.71. The highest range of 0.7236–1 belonged to DC, with a weighted mean of 0.88.

A great scope of intra- and inter-subgroup differences were observed.

In some instances, Se ranged from a practically unsatisfactory 0.6337 to 1, indicating that all lesions were perfectly distinguished as positive, without any FNs. Variations in the amounts of training data, AI systems employed, or study designs serve as plausible explanations for these inconsistencies.

The highest mean values were reported for DC, followed by OKC, AB, and RC. The sample size weighted means for all groups were in the 0.8–0.9 range, which is desirable in the literature.

Two studies measured F1 scores ranging from 0.76 to 0.833. However, direct comparison between these studies is not feasible, as they investigated different pools of lesions rather than individual estimates on both occasions.

Three articles reported AP values. Results for each lesion type were congruent. The overall mean value was 0.73, which implies a need for development.

Our conclusions on detection agree with those of the systematic review by Fedato Tobias et al. [8].

The findings of the two available articles on segmentation present conflicting results. Sivasundaram et al. reported outstanding values for DC, RC, and “odontogenic cyst” groups: Se and Sp were higher than 0.9 [77]. In contrast, Yu et al. indicated that the Se and Sp for DC, RC, AB, and OKC exceeded 0.8. IoU measurements in both studies displayed similar trends [10].

### 4.1. Strengths and Limitations

Following a previously published protocol, this study has transparently summarized all available evidence about AI diagnostics of odontogenic cysts and Abs, established on a rigorous methodology. Each lesion type was investigated and evaluated separately to comprehensively summarize all available classification, detection, and segmentation performance evidence.

Although the whole spectrum of odontogenic cysts was explored in our meta-analysis, a major limitation was the need for further studies. Variations in AI algorithms and study designs (e.g., the number of training images and augmentation) led to higher heterogeneity in some subgroups. This may represent a potential source of bias in the results and therefore warrants careful interpretation.

Image augmentation featured in numerous articles. As this method showed great diversity, non-augmented data were extracted for greater homogeneity and precision where possible. In cases where this was not feasible, information from the augmented group, which typically encompassed a larger population, was included. Consequently, results from the augmented data may be overrepresented in our pooled analysis, which is a limitation of this study.

Researchers have highlighted the dubious applicability of the QUADAS-2 tool for AI and implemented a modified solution, “QUADAS-AI.” As the tool is under development, we followed the recommendations of Cochrane and utilized QUADAS-2. Consequently, caution should be exercised in drawing conclusions [82].

### 4.2. Implications for Practice

Translation of scientific achievements into healthcare is pivotal [83,84]. As a first step, healthcare professionals should be familiarized and trained in the use of novel AI tools. CNNs can be used both as a supplementary option for OPGs in routine dental check-ups or as a prediction tool for the classification of detected cysts.

Leeflang et al. stated that a test could be beneficial as a first-line solution even if the Se or Sp was lower. Therefore, until further development of Se, AI tests can be used under supervision to improve everyday diagnostics [78].

### 4.3. Implications for Research

More robust data is needed for a proper meta-analysis on detection and segmentation performance. The consistently high Sp value across all groups suggests that AI effectively minimizes FP results, reducing unnecessary invasive procedures. However, improvements in sensitivity are necessary to better identify diseased patients.

Future research should also address the diagnostic accuracy of tools integrating multiple modalities (e.g., detection combined with classification), along with a more in-depth examination of ethical considerations.

Furthermore, a more standardized and transparent approach to publication is essential. Providing clear details on AI systems, augmentation techniques, and the number of images used for training, testing, and validation will help accelerate progress in this field.

## 5. Conclusions

AI effectively identifies healthy individuals due to its high specificity. However, its sensitivity for disease detection remains suboptimal and requires further improvement.

## Figures and Tables

**Table 1:** Search strategy.

Search key for PubMed/MEDLINE and Cochrane Central Register of Controlled Trials:
(autom* OR algorithm OR (artificial AND intelligence) OR ai OR (neural AND network) OR convolutional OR cnn OR (deep AND learning) OR ‘machine learning’ OR (computer AND learning) OR ML OR DL) AND (((cyst* OR cyst) AND (dental OR oral OR odonto* OR follicular OR dentigerous OR eruption OR radicular OR periapical OR periodontal OR gingival OR primordial OR keratocyst)) OR ameloblastoma) AND (radiolog* OR imaging OR OP OR x-ray OR panoramic)
Search key for EMBASE:
(autom* OR ‘algorithm’/exp OR algorithm OR (artificial AND (‘intelligence’/exp OR intelligence)) OR ai OR (neural AND (‘network’/exp OR network)) OR convolutional OR cnn OR (deep AND (‘learning’/exp OR learning)) OR ‘machine learning’/exp OR ‘machine learning’ OR ((‘computer’/exp OR computer) AND (‘learning’/exp OR learning)) OR ml OR dl) AND ((cyst* OR ‘cyst’/exp OR cyst) AND (‘dental’/exp OR dental OR oral OR odonto* OR follicular OR dentigerous OR ‘eruption’/exp OR eruption OR radicular OR periapical OR periodontal OR gingival OR ‘primordial’/exp OR primordial OR ‘keratocyst’/exp OR keratocyst) OR ‘ameloblastoma’/exp OR ameloblastoma) AND (radiolog* OR ‘imaging’/exp OR imaging OR op OR ‘x ray’/exp OR ‘x ray’ OR panoramic)

## Data Availability

The original data presented in the study are openly available in the full-text articles included in the systematic review and meta-analysis.

## Supplementary Materials

The following supporting information can be downloaded at https://www.mdpi.com/article/10.3390/jcm15062447/s1: Figure S1: ROC curves for classification performance; Figure S2: Detection results; Figure S3: Risk of bias assessment; Figure S4: Funnel plots; Figure S5: GRADE assessment; Table S1: PRISMA Checklist; Table S2: Baseline Characteristics Table.

## Abbreviations

The following abbreviations are used in this manuscript:

AB	Ameloblastoma
AI	Artificial intelligence
AUC	Area under the receiver operating characteristic curve
CBCT	Cone beam computed tomography
CI	Confidence interval
CNN	Convolutional neural network
DC	Dentigerous cyst
DOR	Diagnostic odds ratio
FN	False negative
FP	False positive
GRADE	Grades of Recommendation, Assessment, Development, and Evaluation
IoU	Intersection over union
N	No lesion
NPDC	Nasopalatine duct cyst
NPV	Negative predictive value
OKC	Odontogenic keratocyst
OPG	Orthopantomography
PIRD	Population, Index test, Reference test, Diagnosis of interest
PPV	Positive predictive value
PRISMA	Preferred Reporting Items for Systematic Reviews and Meta-Analyses
QUADAS	Quality Assessment of Diagnostic Accuracy Studies
RC	Radicular cyst
ROC	Receiver operating characteristic
Se	Sensitivity
Sp	Specificity
TN	True negative
TP	True positive
