# Supplementary material for: Artificial Intelligence in the Diagnosis of Odontogenous Cysts and Ameloblastomas—A Systematic Review and Meta-Analysis
Source: J Clin Med. 2026 Mar 23;15(6):2447. doi: 10.3390/jcm15062447 (PMC13026776; doi:10.3390/jcm15062447)
Supplement: Supplementary file 1 [file jcm-15-02447-s001.zip › Supplementary materials.pdf]

Supplementary materials

Table S1 PRISMA Checklist

Section and Topic	Item #	Checklist item	Location where item is reported
**TITLE**			
Title	1	Identify the report as a systematic review.	1
**ABSTRACT**			
Abstract	2	See the PRISMA 2020 for Abstracts checklist.	1
**INTRODUCTION**			
Rationale	3	Describe the rationale for the review in the context of existing knowledge.	2
Objectives	4	Provide an explicit statement of the objective(s) or question(s) the review addresses.	2
**METHODS**			
Eligibility criteria	5	Specify the inclusion and exclusion criteria for the review and how studies were grouped for the syntheses.	3
Information sources	6	Specify all databases, registers, websites, organisations, reference lists and other sources searched or consulted to identify studies. Specify the date when each source was last searched or consulted.	3
Search strategy	7	Present the full search strategies for all databases, registers and websites, including any filters and limits used.	3, Table 1
Selection process	8	Specify the methods used to decide whether a study met the inclusion criteria of the review, including how many reviewers screened each record and each report retrieved, whether they worked independently, and if applicable, details of automation tools used in the process.	3, 4
Data collection process	9	Specify the methods used to collect data from reports, including how many reviewers collected data from each report, whether they worked independently, any processes for obtaining or confirming data from study investigators, and if applicable, details of automation tools used in the process.	4
Data items	10a	List and define all outcomes for which data were sought. Specify whether all results that were compatible with each outcome domain in each study were sought (e.g. for all measures, time points, analyses), and if not, the methods used to decide which results to collect.	4
	10b	List and define all other variables for which data were sought (e.g. participant and intervention characteristics, funding sources). Describe any assumptions made about any missing or unclear information.	4
Study risk of bias assessment	11	Specify the methods used to assess risk of bias in the included studies, including details of the tool(s) used, how many reviewers assessed each study and whether they worked independently, and if applicable, details of automation tools used in the process.	4
Effect measures	12	Specify for each outcome the effect measure(s) (e.g. risk ratio, mean difference) used in the synthesis or presentation of results.	4
Synthesis methods	13a	Describe the processes used to decide which studies were eligible for each synthesis (e.g. tabulating the study intervention characteristics and comparing against the planned groups for each synthesis (item #5)).	4, 5
	13b	Describe any methods required to prepare the data for presentation or synthesis, such as handling of missing summary statistics, or data	4, 5

Section and Topic	Item #	Checklist item	Location where item is reported
		conversions.	
	13c	Describe any methods used to tabulate or visually display results of individual studies and syntheses.	4, 5
	13d	Describe any methods used to synthesize results and provide a rationale for the choice(s). If meta-analysis was performed, describe the model(s), method(s) to identify the presence and extent of statistical heterogeneity, and software package(s) used.	4, 5
	13e	Describe any methods used to explore possible causes of heterogeneity among study results (e.g. subgroup analysis, meta-regression).	4, 5
	13f	Describe any sensitivity analyses conducted to assess robustness of the synthesized results.	4, 5
Reporting bias assessment	14	Describe any methods used to assess risk of bias due to missing results in a synthesis (arising from reporting biases).	4, 5
Certainty assessment	15	Describe any methods used to assess certainty (or confidence) in the body of evidence for an outcome.	5
**RESULTS**			
Study selection	16a	Describe the results of the search and selection process, from the number of records identified in the search to the number of studies included in the review, ideally using a flow diagram.	5, 6 Figure 1
	16b	Cite studies that might appear to meet the inclusion criteria, but which were excluded, and explain why they were excluded.	5, 6 Figure 1
Study characteristics	17	Cite each included study and present its characteristics.	5-11, Table S2
Risk of bias in studies	18	Present assessments of risk of bias for each included study.	11, Figure S3
Results of individual studies	19	For all outcomes, present, for each study: (a) summary statistics for each group (where appropriate) and (b) an effect estimate and its precision (e.g. confidence/credible interval), ideally using structured tables or plots.	5-11, Figures 2-6, Figure S2
Results of syntheses	20a	For each synthesis, briefly summarise the characteristics and risk of bias among contributing studies.	10, Figure S3, Table S2
	20b	Present results of all statistical syntheses conducted. If meta-analysis was done, present for each the summary estimate and its precision (e.g. confidence/credible interval) and measures of statistical heterogeneity. If comparing groups, describe the direction of the effect.	5-11, Figures 2-6
	20c	Present results of all investigations of possible causes of heterogeneity among study results.	5-11
	20d	Present results of all sensitivity analyses conducted to assess the robustness of the synthesized results.	11
Reporting biases	21	Present assessments of risk of bias due to missing results (arising from reporting biases) for each synthesis assessed.	11
Certainty of evidence	22	Present assessments of certainty (or confidence) in the body of evidence for each outcome assessed.	11, Figure S5
**DISCUSSION**			
Discussion	23a	Provide a general interpretation of the results in the context of other evidence.	11, 12

Section and Topic	Item #	Checklist item	Location where item is reported
	23b	Discuss any limitations of the evidence included in the review.	13
	23c	Discuss any limitations of the review processes used.	13
	23d	Discuss implications of the results for practice, policy, and future research.	13
**OTHER INFORMATION**			
Registration and protocol	24a	Provide registration information for the review, including register name and registration number, or state that the review was not registered.	3
	24b	Indicate where the review protocol can be accessed, or state that a protocol was not prepared.	3
	24c	Describe and explain any amendments to information provided at registration or in the protocol.	3
Support	25	Describe sources of financial or non-financial support for the review, and the role of the funders or sponsors in the review.	14
Competing interests	26	Declare any competing interests of review authors.	14
Availability of data, code and other materials	27	Report which of the following are publicly available and where they can be found: template data collection forms; data extracted from included studies; data used for all analyses; analytic code; any other materials used in the review.	14

From: Page MJ, McKenzie JE, Bossuyt PM, Boutron I, Hoffmann TC, Mulrow CD, et al. The PRISMA 2020 statement: an updated guideline for reporting systematic reviews. BMJ 2021;372:n71. doi: 10.1136/bmj.n71. This work is licensed under CC BY 4.0. To view a copy of this license, visit https://creativecommons.org/licenses/by/4.0/

Figure S1 ROC curves for classification performance

a AB ROC curve

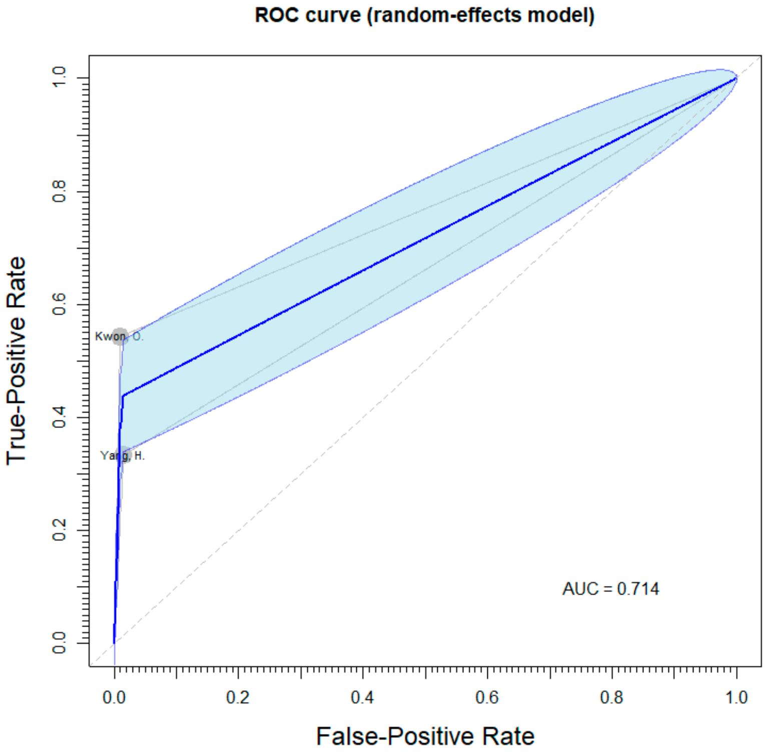

b DC ROC curve

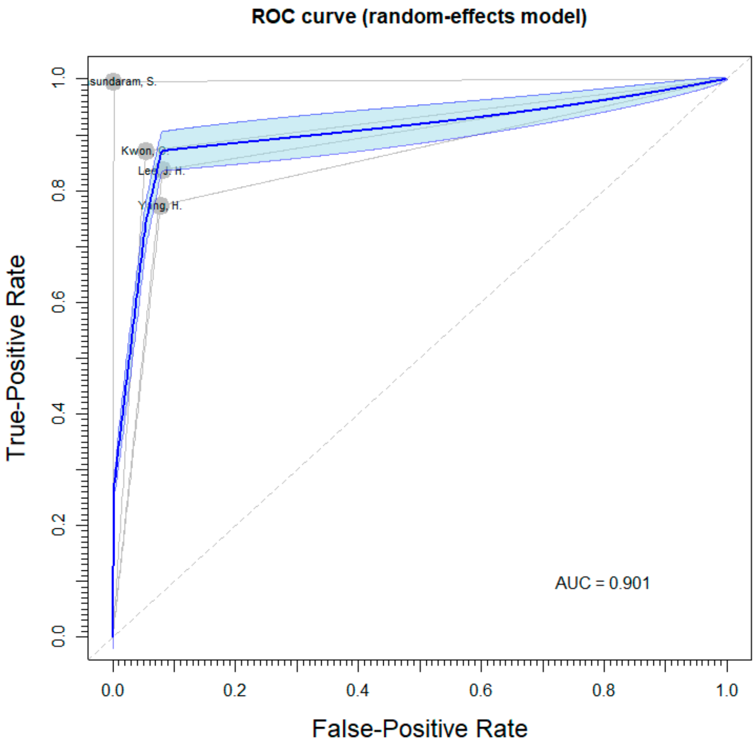

c N ROC curve

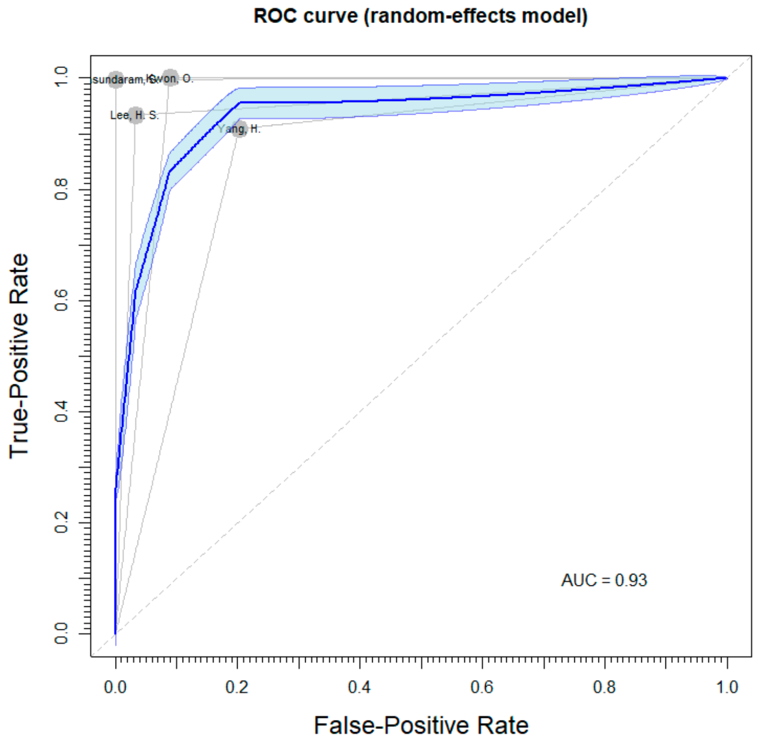

d OKC ROC curve

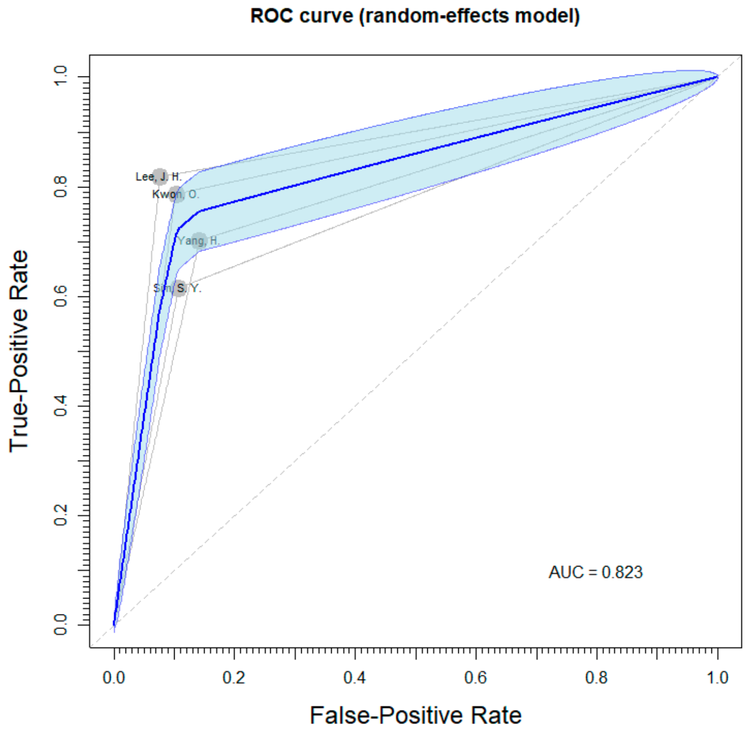

e RC ROC curve

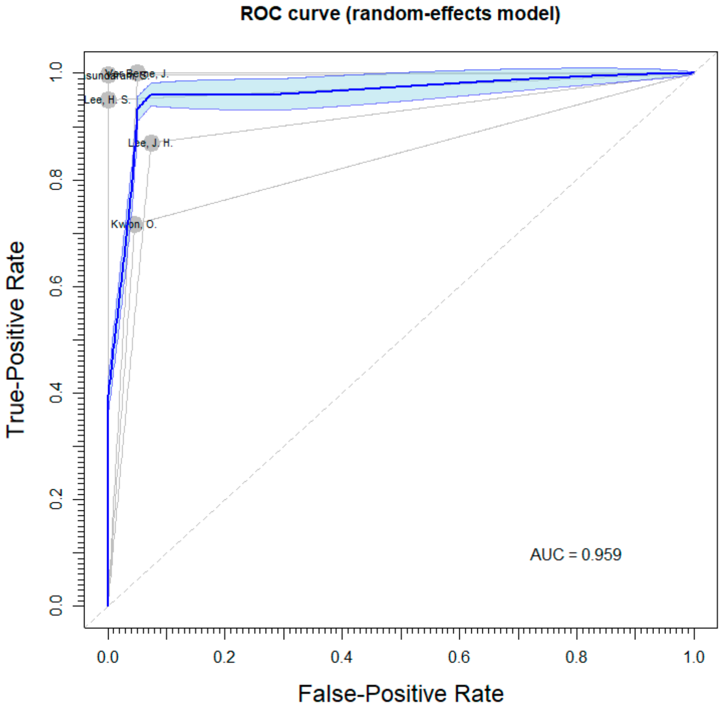

Figure S2 Detection results

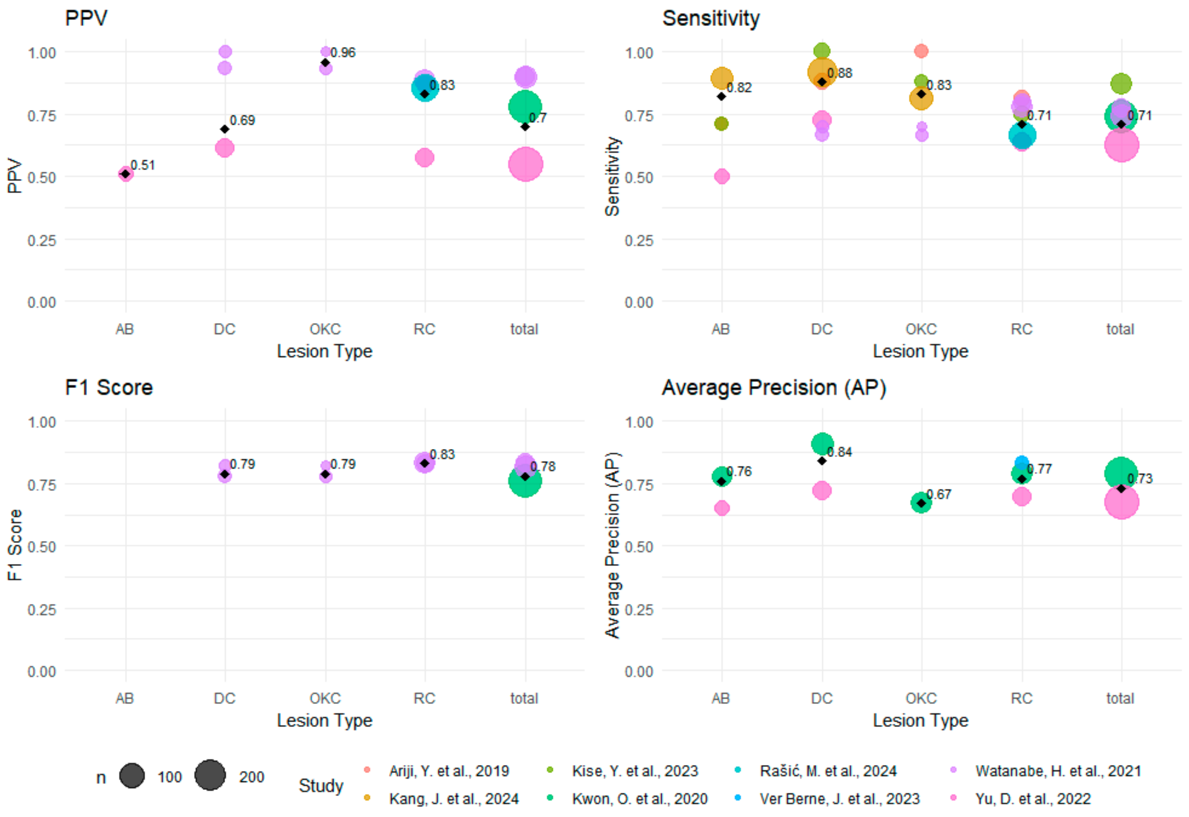

Figure S3

a Risk of bias assessment (QUADAS-2 – Risk of

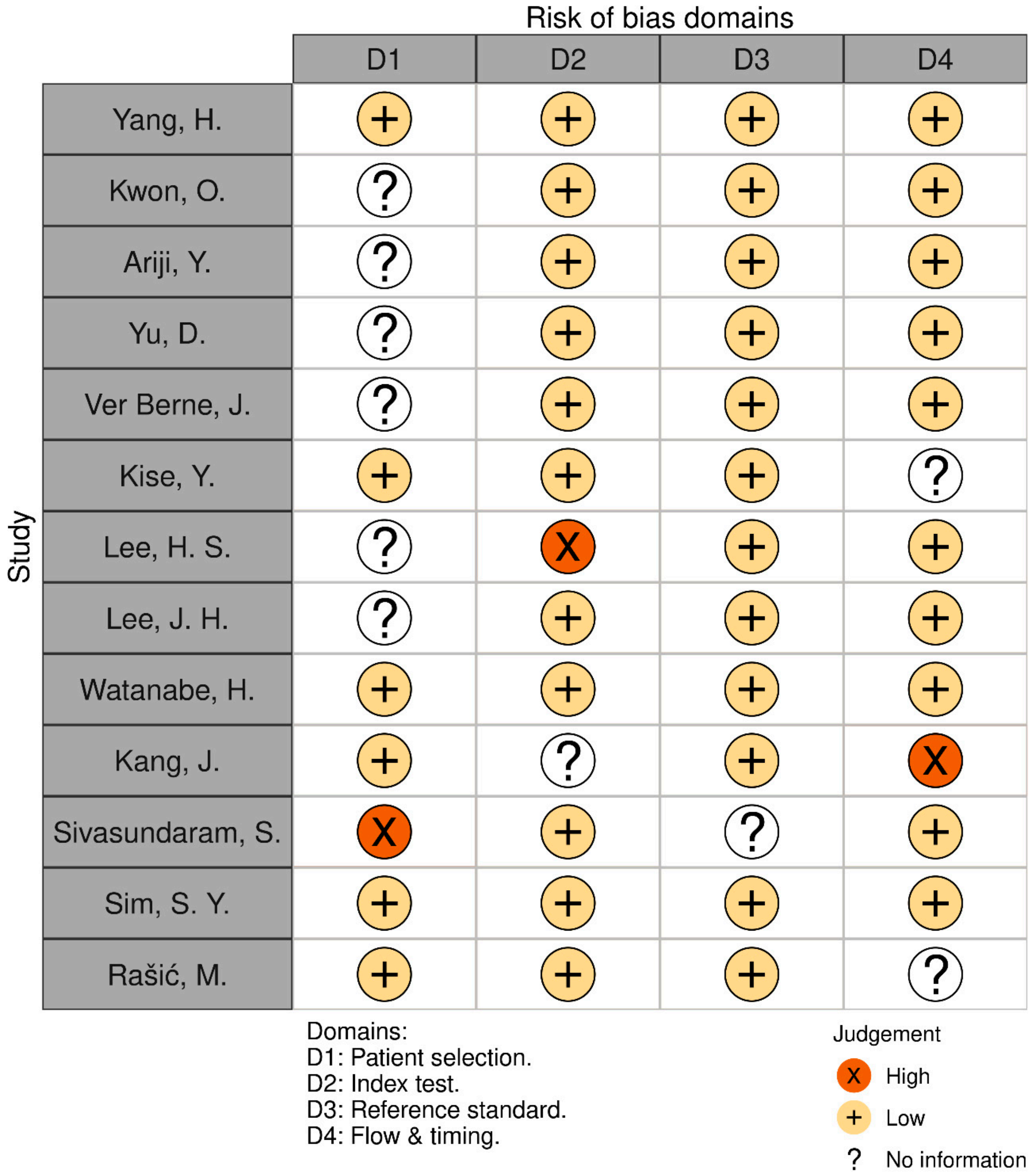

bias)

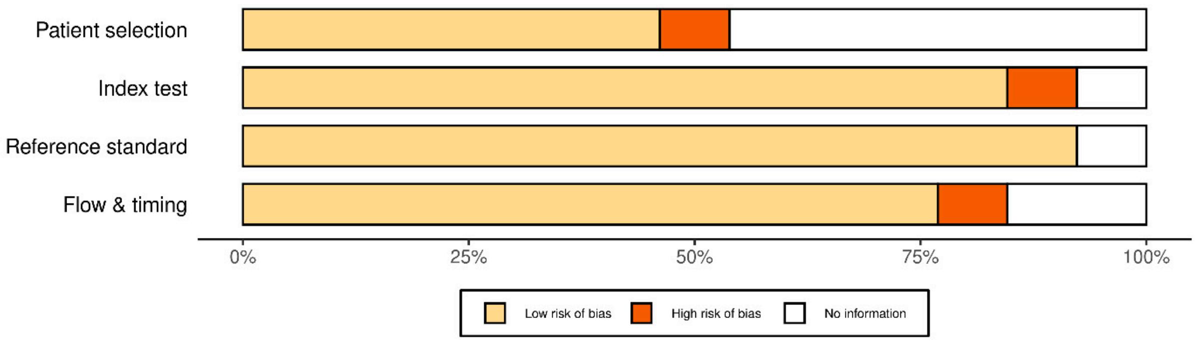

b Risk of bias assessment (QUADAS-2 – Applicability concerns)

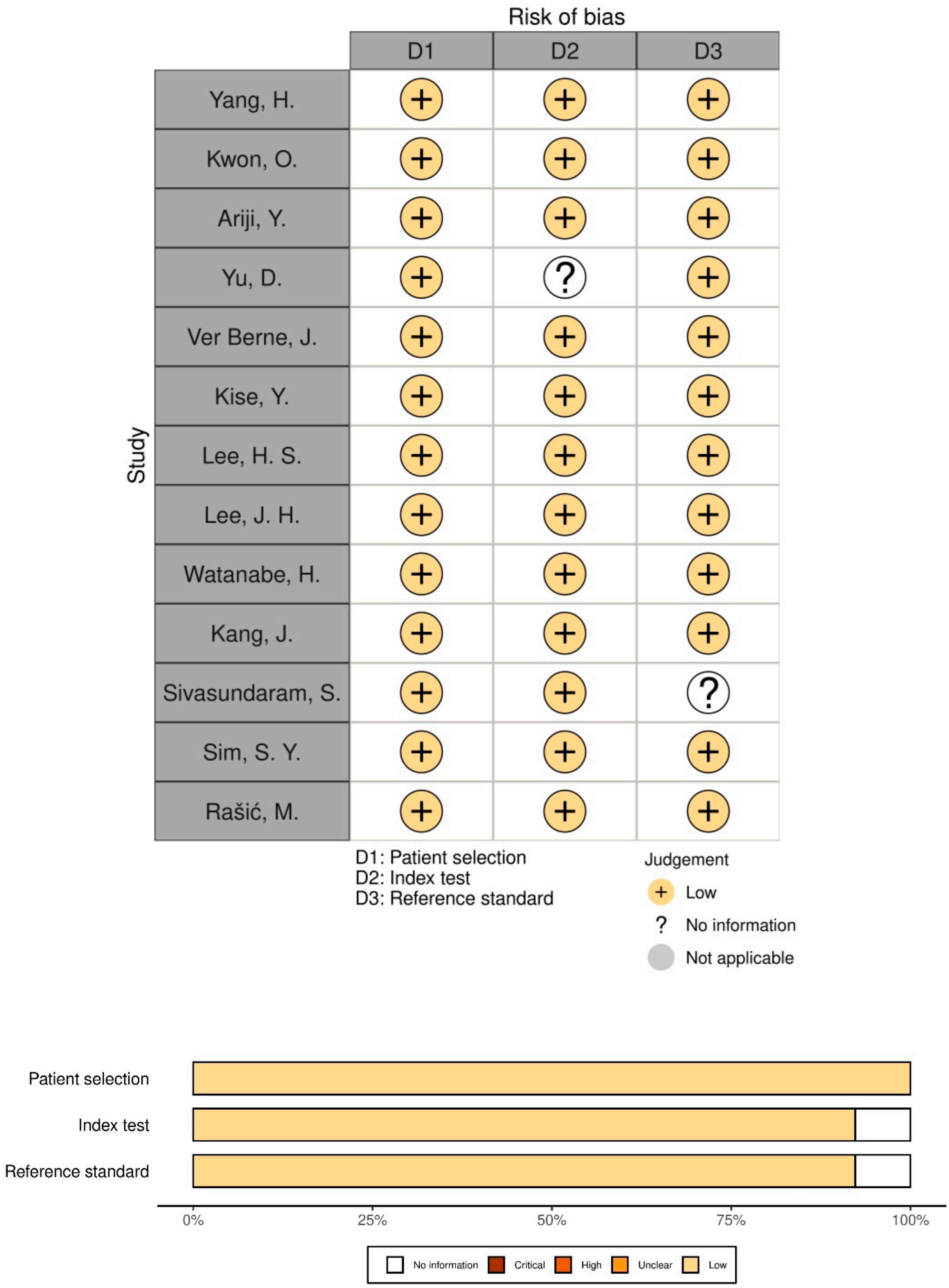

Figure S4

a Funnel plot for AB classification sensitivity

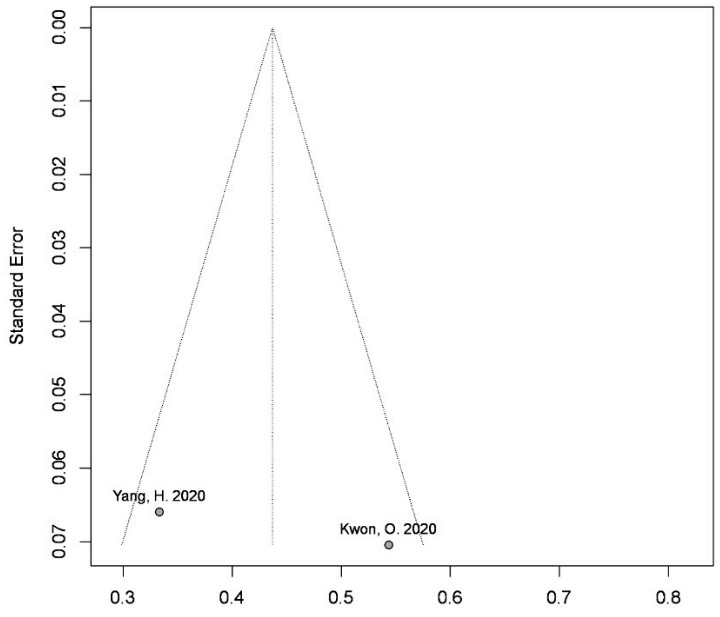

b Funnel plot for DC classification sensitivity

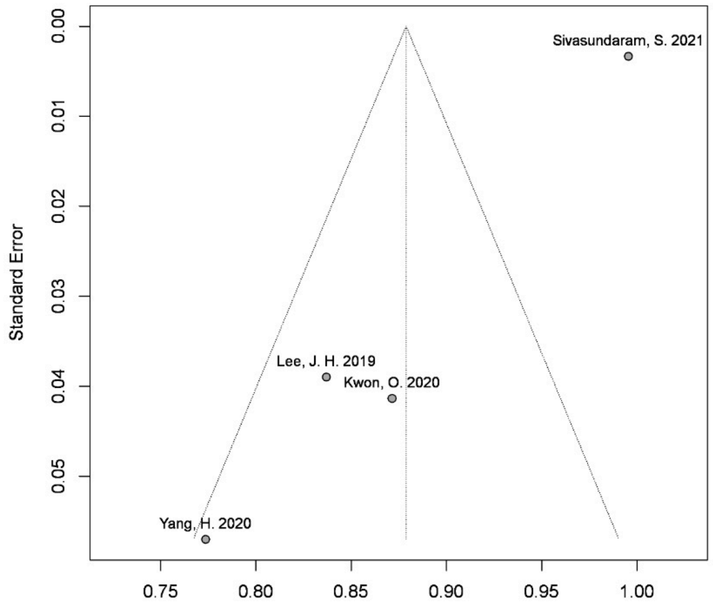

c Funnel plot for N classification sensitivity

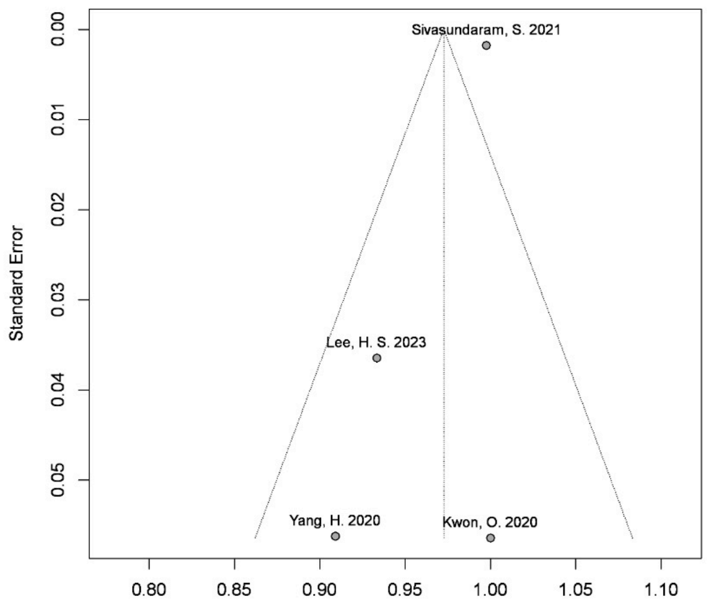

d Funnel plot for OKC classification sensitivity

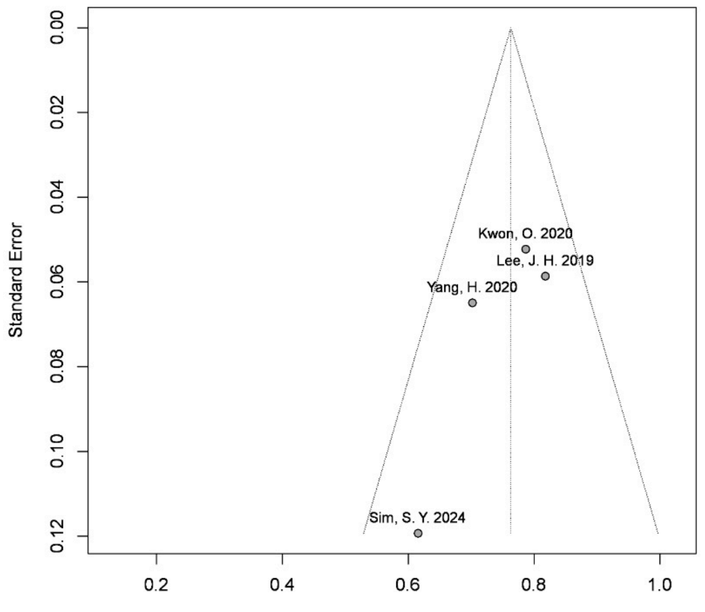

e Funnel plot for RC classification sensitivity

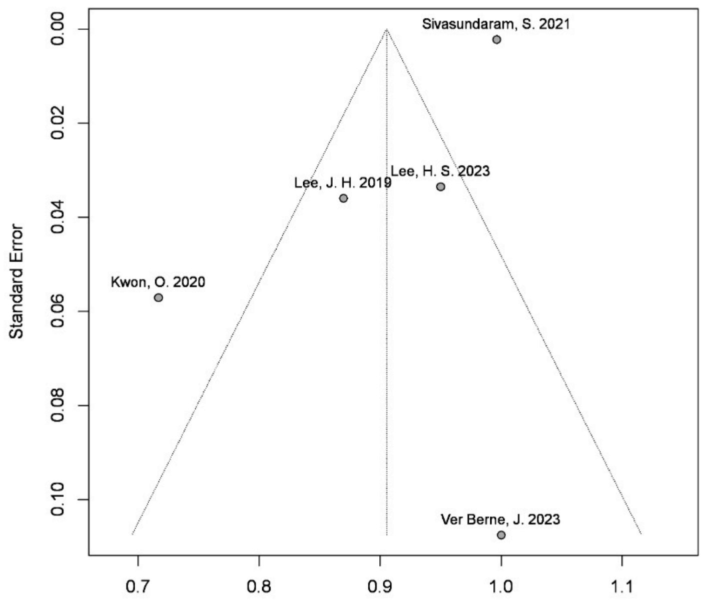

f Funnel plot for AB classification specificity

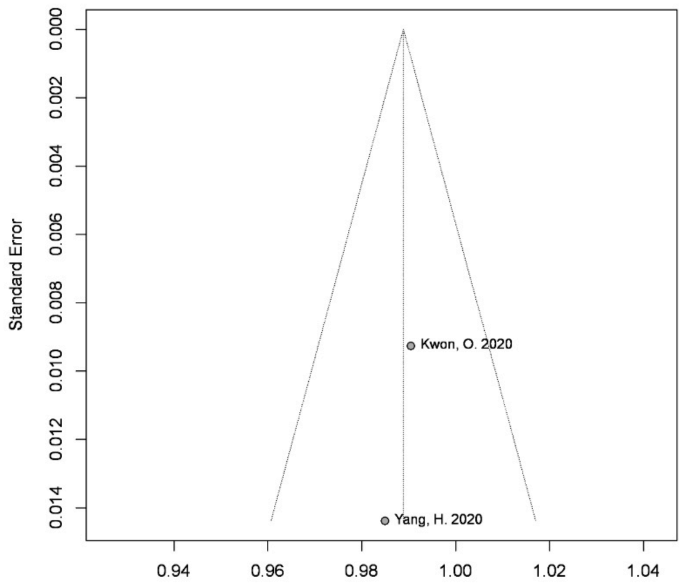

g Funnel plot for DC classification specificity

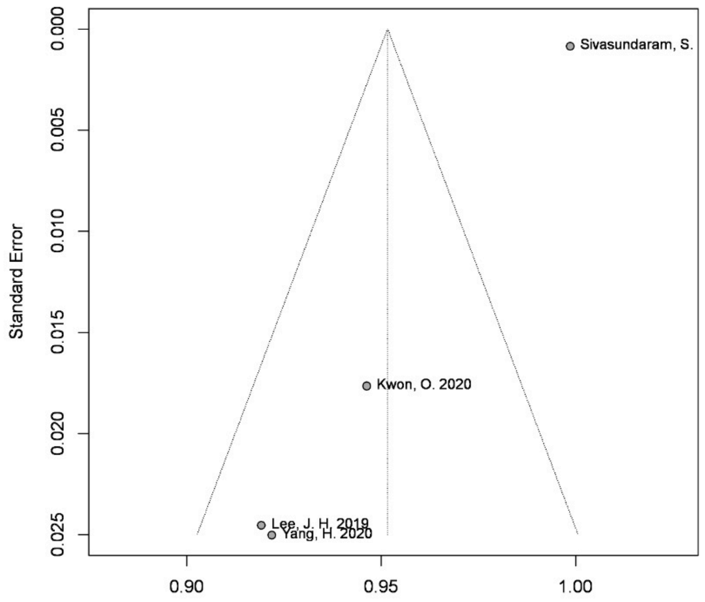

h Funnel plot for N classification specificity

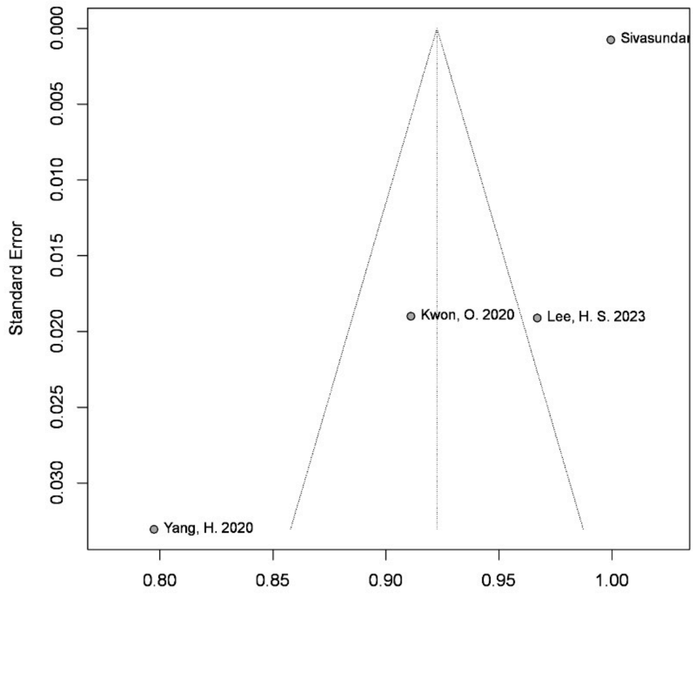

i Funnel plot for OKC classification specificity

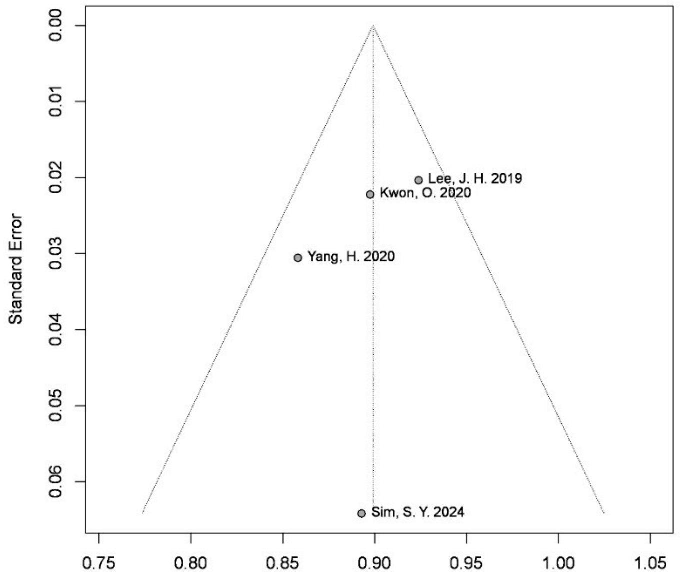

j Funnel plot for RC classification specificity

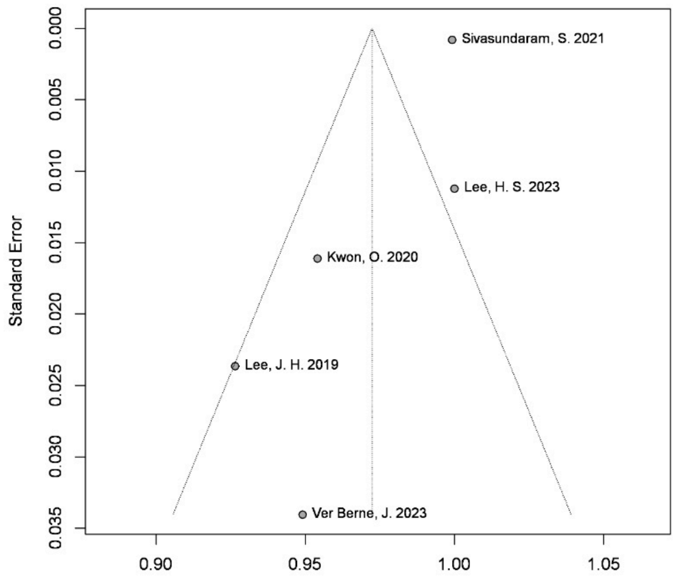

Figure S5 GRADE assessment

Question: Should artificial intelligence be used to diagnose ameloblastomas in patients with panoramic radiographs?

Sensitivity	0.44 (95% CI: 0.23 to 0.64)
Specificity	0.99 (95% CI: 0.97 to 1.00)

Outcome	№ of studies (№ of patients)	Study design	Factors that may decrease certainty of evidence					Effect per 1000 patients tested	Test accuracy CoE		
			Risk of bias	Indirectness	Inconsistency	Imprecision	Publication bias	pre-test probability of0%			
**True positives** (patients with ameloblastomas)	2 studies 94 patients	cohort & case-control type studies	not serious	not serious	serious	very serious[a]	none	0 (0 to 0)			⊕◯◯◯ Very low[a]
**False negatives** (patients incorrectly classified as not having ameloblastomas)								0 (0 to 0)			
**True negatives** (patients without ameloblastomas)	2 studies 343 patients	cohort & case-control type studies	not serious	not serious	serious[b]	very serious[a]	none	989 (974 to 1000)			⊕◯◯◯ Very low[a,b]
**False positives** (patients incorrectly classified as having ameloblastomas)								11 (0 to 26)			

Explanations

a. Low number of patients and studies

b. High heterogeneity

Question: Should artificial intelligence be used to diagnose dentigerous cysts in patients with panoramic radiographs?

Sensitivity	0.88 (95% CI: 0.78 to 0.97)
Specificity	0.95 (95% CI: 0.91 to 0.99)

Outcome	№ of studies (№ of patients)	Study design	Factors that may decrease certainty of evidence					Effect per 1000 patients tested	Test accuracy CoE		
			Risk of bias	Indirectness	Inconsistency	Imprecision	Publication bias	pre-test probability of0%			
**True positives** (patients with dentigerous cysts)	4 studies 875 patients	cohort & case-control type studies	not serious	not serious	serious	serious[a]	strong association	0 (0 to 0)			⊕⊕⊕◯ Moderate[a]
**False negatives** (patients incorrectly classified as not having dentigerous cysts)								0 (0 to 0)			
**True negatives** (patients without dentigerous cysts)	4 studies 3303 patients	cohort & case-control type studies	not serious	not serious	serious[b]	serious	strong association	952 (912 to 992)			⊕⊕⊕◯ Moderate[b]
**False positives** (patients incorrectly classified as having dentigerous cysts)								48 (8 to 88)			

Explanations

a. Low number of patients and studies

b. High heterogeneity and the difference in the presence of image augmentation.

Question: Should artificial intelligence be used to diagnose odontogenic keratocysts in patients with panoramic radiographs?

Sensitivity	0.76 (95% CI: 0.70 to 0.83)
Specificity	0.90 (95% CI: 0.87 to 0.93)

Outcome	№ of studies (№ of patients)	Study design	Factors that may decrease certainty of evidence					Effect per 1000 patients tested	Test accuracy CoE		
			Risk of bias	Indirectness	Inconsistency	Imprecision	Publication bias	pre-test probability of0%			
**True positives**<br>(patients with odontogenic keratocysts)	4 studies<br>165 patients	cohort & case-control type studies	not serious	not serious	serious	serious[a]	strong association	0 (0 to 0)			⨁⨁⨁◯<br>Moderate[a]
**False negatives**<br>(patients incorrectly classified as not having odontogenic keratocysts)								0 (0 to 0)			
**True negatives**<br>(patients without odontogenic keratocysts)	4 studies<br>541 patients	cohort & case-control type studies	not serious	not serious	serious[b]	serious	strong association	899 (868 to 930)			⨁⨁⨁◯<br>Moderate[b]
**False positives**<br>(patients incorrectly classified as having odontogenic keratocysts)								101 (70 to 132)			

Explanations

a. Low number of patients and studies

b. High heterogeneity

Question: Should artificial intelligence be used to diagnose radicular cysts in patients with panoramic radiographs?

Sensitivity	0.91 (95% CI: 0.81 to 1.00)
Specificity	0.97 (95% CI: 0.94 to 1.00)

Outcome	№ of studies (№ of patients)	Study design	Factors that may decrease certainty of evidence					Effect per 1000 patients tested	Test accuracy CoE		
			Risk of bias	Indirectness	Inconsistency	Imprecision	Publication bias	pre-test probability of0%			
**True positives** (patients with radicular cysts)	5 studies 1289 patients	cohort & case-control type studies	not serious	not serious	serious	serious[a]	strong association	0 (0 to 0)			⊕⊕⊕◯ Moderate[a]
**False negatives** (patients incorrectly classified as not having radicular cysts)								0 (0 to 0)			
**True negatives** (patients without radicular cysts)	5 studies 2956 patients	cohort & case-control type studies	not serious	not serious	serious[b]	serious	strong association	972 (943 to 1000)			⊕⊕⊕◯ Moderate[b]
**False positives** (patients incorrectly classified as having radicular cysts)								28 (0 to 57)			

Explanations

a. Low number of patients and studies

b. High heterogeneity

Question: Should artificial intelligence be used to diagnose no lesion in patients with panoramic radiographs?

Sensitivity	0.97 (95% CI: 0.93 to 1.00)
Specificity	0.92 (95% CI: 0.84 to 1.00)

Prevalences	0%		

Outcome	№ of studies (№ of patients)	Study design	Factors that may decrease certainty of evidence					Effect per 1000 patients tested	Test accuracy CoE		
			Risk of bias	Indirectness	Inconsistency	Imprecision	Publication bias	pre-test probability of0%			
**True positives**<br>(patients with no lesion)	4 studies<br>1371 patients	cohort & case-control type studies	not serious	not serious	serious	serious[a]	strong association	0 (0 to 0)			⊕⊕⊕◯<br>Moderate[a]
**False negatives**<br>(patients incorrectly classified as not having no lesion)								0 (0 to 0)			
**True negatives**<br>(patients without no lesion)	4 studies<br>2760 patients	cohort & case-control type studies	not serious	not serious	serious[b]	serious	strong association	923 (839 to 1000)			⊕⊕⊕◯<br>Moderate[b]
**False positives**<br>(patients incorrectly classified as having no lesion)								77 (0 to 161)			

Explanations

a. Low number of patients and studies

b. High heterogeneity
